# Stability of, and Associations Between, Parent and Child Locus of Control Expectancies

**DOI:** 10.3389/fpsyg.2018.02018

**Published:** 2018-10-25

**Authors:** Stephen Nowicki, Yasmin Iles-Caven, Steven Gregory, Genette Ellis, Jean Golding

**Affiliations:** ^1^Department of Psychology, Emory University, Atlanta, GA, United States; ^2^Centre for Academic Child Health, Population Health Sciences, Bristol Medical School, University of Bristol, Bristol, United Kingdom

**Keywords:** ALSPAC, longitudinal cohort, parental locus of control, child locus of control, adolescent locus of control, stability over time, Rotter’s concept of locus of control

## Abstract

The purpose of the present study was to assess the stability of locus of control (LOC) scores over time using data gathered from tests constructed to be consistent with Rotter’s definition of LOC. We compared LOC scores of parents (measured prior to the birth of the index child and at 6 and 18 years later) and their offspring (at ages 8 and 16) to explore how stable adult and child LOC was over time and to see how parental LOC was associated with the LOC of the child aged 8 and again at 16. Locus of control was measured using modified versions of adult (ANSIE, Nowicki and Duke, 1974) and child (CNSIE, Nowicki and Strickland, 1973) LOC scales, administered to participants in the Avon Longitudinal Study of Parents and Children in the United Kingdom. We predicted that: (1) adult scores would be more stable than children’s and (2) parents’ and children’s LOC scores would be related to one another. Analyses of the data found that individual’s LOC scores were significantly associated over time, with adult scores (*r* ∼ 0.50) more highly correlated than children’s (*r* ∼ 0.20). Correlations suggest more stability for adults than children, but also indicate the occurrence of substantial change across time. Although statistically significant, correlations between family members were small at both childhood and adolescent time points. Additional analyses suggested that mother and father LOC scores were more highly correlated with opposite rather than with same sex children, but again though significant the coefficients were small. We also analyzed the binary outcomes of externality to assess parental contributions to externality in the 8 and 16-year-old children and found correlations were significant, but small. Possible explanations are offered for why the associations between parent and child LOC were not higher. We concluded that researchers need to focus more on clarifying how children’s LOC is acquired.

## Introduction

Over a half century ago, Rotter (1966) introduced the concept of locus of control of reinforcement (LOC) to the psychological literature defining it as a generalized problem-solving expectancy functioning within his social learning theory. Within that now classic article, Rotter also presented a test to measure the degree to which reinforcement outcomes were perceived to be related to behavior. He defined LOC as follows: “Internal versus external control refers to the degree to which persons expect that a reinforcement or an outcome of their behavior is contingent on their own behavior or personal characteristics versus the degree to which persons expect that the reinforcement or outcome is a function of chance, luck, or fate, is under the control of powerful others, or is simply unpredictable. Such expectancies may generalize along a gradient based on the degree of semantic similarity of the situational cues” (p. 1).

By Rotter’s definition, LOC is a *generalized expectancy* which means that it varies in its impact as a function of an individual’s greater or lesser experience across and within situations. As a generalized expectancy, LOC is assumed to have its maximum impact on behavior when individuals have little or no experience in the situation or when the situation is ambiguous, amorphous, or fluid. As experience is gained from being in a specific situation, the ability of a generalized expectancy, such as LOC, to affect behavior diminishes and specific expectancies learned from being in the situation become more important. However, should a situation change and therefore become “new” again (as for example when a company is going through a management transition or when a child faces a change in teachers in school) then generalized expectancies may once again become an important predictor of behavior.

We emphasize Rotter’s unique definition of LOC because, since he introduced it, researchers have employed various terms interchangeably with Rotter’s, or used “locus of control” to refer to constructs other than the one offered by Rotter. In fact, 30 years after Rotter’s article, not only did Skinner (1996) find that Rotter’s LOC of reinforcement term had been shortened to simply “control,” but that over a 100 different terms had been used to describe it such as: “…personal control, sense of control, LOC, cognitive control, agenda control, outcome control, primary control, secondary control, action control, decisional control, predictive control, informational control and proxy control….” Moreover within the total set of terms, some appear to be different labels for the same construct. For example, Bandura (1977) referred to “a personal estimate that a given behavior will lead to a certain outcome” (p. 193) as “response-outcome expectancies,” whereas Heckhausen (1977) labeled the subjective probably that one’s actions will modify a situation “action-outcome expectancy,” and Seligman (1975) described the degree of the relationship between responses and outcomes in terms of “contingencies.”

Although two decades have passed since Skinner’s comprehensive review, Nowicki and Duke (2016) noted there was still confusion about what is and what is not LOC. They suggested some of the confusion may be the result of what Kelley (1927) called “jingle” and “jangle” fallacies in the presentation of concepts. In the “jingle fallacy” a single term is used to describe different things. For example, researchers use “locus of control” but define it differently than Rotter’s description of a generalized expectancy (Lachman and Weaver, 1998; Cobb-Clark and Schurer, 2013). In contrast the “jangle fallacy” occurs when a different term such as “sense” of control is used synonymously with LOC (e.g., Ahlin and Lobo Antunes, 2015).

To the two conceptual fallacies offered by Kelly, we add a third, “the jumble fallacy” in which researchers use constructs like self-efficacy (Bandura, 1977) and attribution (Seligman, 1975), interchangeably with Rotter’s LOC construct. Although Peterson and Stunkard (1992) have clearly pointed out the conceptual differences among the three constructs, many researchers still switch efficacy, attribution, and LOC with one another as though they are referring to the same construct which they are not.

### Problems in the Measurement of Locus of Control

Compounding the confusion created by the existence of so many LOC terms are the difficulties originating in the 100s of different tests used to measure them. Unfortunately, there is not a review of LOC tests comparable to the one Skinner completed for “terms.” Part of the measurement problem is that researchers often do not present construct validity evidence for their LOC test as Rotter did with his (see Cronbach and Meehl, 1955). Rotter (1966) used data from many studies to establish “preliminary” construct validity for his definition of LOC. As suggested by Cronbach and Meehl, Rotter built his test by sampling items from the universe of items consistent with his definition of the construct and then provided evidence to accept or reject associations predicted by it.

Unfortunately, other LOC researchers have not always been as thorough in presenting evidence of their tests’ construct validity. In one case, a single test item was used (Conell-Price and Jamison (2015). In other instances, items used to measure LOC were taken from tests constructed to measure other constructs such as “coping” (e.g., Cobb-Clark and Schurer, 2013) and were not accompanied by any other construct validity support aside from internal consistency estimates. Rarely do researchers present test data revealing the relationship with Rotter’s LOC test or any other test of “locus of control.”

In summary, past researchers have not always made it clear how their control conceptualizations and the tests used to assess them relate to Rotter’s initial construct or with one another. This makes it difficult to assess how results using one test generalize to those found with others.

Pertinent to this point, Twenge et al. (2004) collected Rotter’s test results for adults and Nowicki and Strickland findings for children over a 30-year period and reported that scores became more external over time. Some years later, Trzesniewski and Donnellan (2010) using data from another 30-year study of high school seniors concluded that there were no changes toward externality over time. However, the authors used a seven-item scale with unknown construct validity to reach their conclusion in contrast to Twenge, Zhang, and Im who used scales with known construct validity. The lack of convergent and discriminative evidence of the validity of tests makes it difficult to know how findings of different studies relate to one another.

Because of the confusion caused by different conceptualizations of LOC and the paucity of construct validity data of tests employed to assess them, in the present study we return to Rotter’s original definition of LOC and use data collected from tests developed consistent with his definition. Much previous research gathered about the stability of individuals’ LOC over time was gathered from cross-sectional studies using small numbers of non-representative participants. There are exceptions. The British Child Health and Education Study (CHES) was a large cohort study that included participants from the United Kingdom born during a 1 week (Elliott and Shepherd, 2006). An Anglicized form of the Nowicki and Strickland test was administered to children aged 10. Scores predicted adult outcomes regarding obesity, blood pressure, and educational attainment (Flouri, 2006; Gale et al., 2008). The test was re-administered when children were 16, and Furnham and Chen (2016) found that childhood intelligence, self-esteem, neuroticism, and earlier child adjustment were antecedents associated with LOC at this age. However, no attempt was made to assess LOC stability between ages 10 and 16.

As mentioned above, Twenge et al. (2004) found that both adults and children became more externally controlled over time. However, the data were cross-sectional and did not follow individuals over time. We could find only two studies that met the criteria of being longitudinal and used tests consistent with Rotter’s definition: Schneewind (1997) and Lekfuangfu et al. (2017).

Schneewind had parents and children take LOC tests as part of a longitudinal study of family relationships. The initial test took place when children were age 10 and parents’ mean age was 36. Sixteen years later Schneewind was able to retest 100 parents and their children. He found that over the 16 years, parent LOC correlated in the 0.50s while children’s scores were in the 0.20s. In addition, parent/child LOC associations, though significant, were small.

Lekfuangfu et al. (2017) used a larger cohort than Schneewind, but also used Anglicized versions of the Nowicki and Strickland LOC tests. They focused on mother’s prenatal LOC and subsequent parenting and child outcomes. Using an economic perspective in which subjective belief in control (LOC) was conceptualized to reflect the degree of investment in child outcomes, they found that prenatal maternal internality was associated with more effective parent attitudes that in turn were related to more time and attention to children and better achievement outcomes. The researchers did not include fathers or other time periods.

### The Present Study

In the present study we use tests constructed to be consistent with Rotter’s concept to examine the stability of adult LOC measured before the child was born (mean maternal age 26; paternal age 29) and measured again when the child was age 6 (adult mean parental ages 32 and 35) and 18–20 (parental mean ages 44 and 49). In addition, we assess the stability of children’s locus of score between the ages of 8 and 18.

No previous study has included both mothers’ and fathers’ LOC obtained prenatally and 6 and 18–20 years later as well as measures of their children’s LOC during childhood and adolescence. This structure not only allows us to examine the stability across time of different age groups, but also to assess the associations between children and their parents during childhood and adolescence. Previous information regarding the stability or change of LOC as measured by Rotter defined LOC tests during a life time has primarily come from cross-sectional studies.

### Stability of Locus of Control: Children

We are tracking the generalized LOC of reinforcement across significant developmental periods for children and adults. When developing the children’s Nowicki and Strickland LOC scale the authors stated outcomes needed for the scale to obtain preliminary construct validity: (1) scores will become more internal with increasing age, (2) scores will be related to achievement with internals achieving more than externals, (3) scores will not be related to measures of social desirability, (4) scores will be related to scores from other tests of LOC. Support was found for the predicted outcomes (see section “Materials and Methods” for more specific construct validity information).

There are significant psychological and physiological changes that take place between age 8 and age 16 that may, in turn, produce changes in LOC. In reality, because of their increasing physical, cognitive and psychological maturation, children gain more control over outcomes with time. Cross-sectional data suggest children become more internal with age, but do not tell us if that is what takes place within individuals as they move from childhood into adolescence. Schneewind (1997) found correlations in the 0.20s between children’s LOC scores at age 10 and their scores at age 26.

### Stability of Locus of Control: Adults

The Adult Nowicki and Strickland Internal-External control scale (ANSIE, Nowicki and Duke, 1974) was constructed by modification of the children’s scale by changing the word “children” to “people” (*n* = 6) and the present tense to the past tense (*n* = 5). This was done to provide a LOC scale for non-college as well as college adults that was consistent with Rotter’s definition, but with an easier reading level. More detail about the ANSIE is presented in the measures section.

Typical adults continue to change toward internality until they reach older age (Nowicki, 2018a, unpublished^1^) and some theorists (e.g., Lachman, 2015) have described adults as going through developmental life changes as do children. However, typical changes in adults wouldn’t be as large as they would be with children and should result in more stable LOC expectancies. Schneewind found correlations in the 0.50s between LOC of adults when their children were age 10 and when their children were age 26. However, he did not test adults before children were born and therefore could not assess the impact the birth of a child could have on their LOC.

### Locus of Control Associations Among Mother, Father, Son, and Daughter

There is surprising little research upon which to base predictions for the LOC associations within a family (see Ollendick, 1979). Only one previous study (Schneewind, 1997) included assessment of fathers as well as mothers and he found correlations between mothers’ and fathers’ LOC were higher than they were between either of them and their child. Because of a lack of past empirical research, no predictions were made for within family associations.

## Materials and Methods

### Participants

The Avon Longitudinal Study of Parents and Children (ALSPAC) was designed to determine the environmental and genetic factors that are associated with the health and development of the study offspring (Golding and The ALSPAC Study Team, 2004; Boyd et al., 2013; Fraser et al., 2013). As part of the study design there was a concerted effort to obtain baseline details on parents’ personalities, moods and attitudes, including a measure of their LOC, prior to the baby’s birth. ALSPAC recruited 14,541 pregnant women residing in Avon, an area of south-west England, with expected dates of delivery between 1st April 1991 and 31st December 1992. Of these initial pregnancies, there was a total of 14,676 fetuses, resulting in 14,062 live births, 13,988 of whom were alive at 1 year of age. Data were collected at various time-points using self-completion questionnaires, biological samples, hands-on measurements, teacher reports and linkage to other data sets. Please note that the study website contains details of all the data that is available through a fully searchable data dictionary and variable search tool: http://www.bristol.ac.uk/alspac/researchers/our-data/.

The mothers and offspring have been followed throughout, but partners were only included initially with the permission of the mothers. Mothers were given a questionnaire which they could pass to their partner if they wished him to participate; partners were given their own reply-paid envelope in which to return their completed questionnaires to avoid potential bias and protect confidentiality.

Ethical approval for the study was obtained from the ALSPAC Ethics and Law Committee [ALEC; IRB00003312] (registered on the Office of Human Research Protections database as U Bristol IRB #1), and the Local Research Ethics Committees. The Committees agreed that consent was implied if questionnaires were returned, and informed written consent was obtained from all participants who underwent certain invasive procedures undertaken during the hands-on assessments (which were optional to attend), and for all biological samples prior to analysis (see Birmingham, 2018).

### Measures

#### Adult Nowicki and Strickland Internal External Control (ANSIE Nowicki and Duke, 1974)

Parents completed an abbreviated version of the ANSIE (Nowicki and Duke, 1974). The ANSIE was an upward extension of the Children’s Nowicki and Strickland Internal-External scale (CNSIE, Nowicki and Strickland, 1973) that was constructed to meet the need for an easier to read version of Rotter’s LOC scale that could be given to community samples. In the initial stages of construct validation both the Rotter and ANSIE scales were given to samples of adults and found to be significantly correlated with one another (*r* = 0.42). Nowicki and Duke (1974) report split-half reliabilities in the 0.60s for college (*n* = 156) and community samples (*n* = 33). These split-half reliabilities seem to be satisfactory in light of the fact that these personality items are not arranged according to difficulty. This makes the split-half reliabilities an underestimate of the true internal consistency reliability (others have reported KR-20s in the 0.60s; Nowicki, 2018a, unpublished^1^). Factor analyses suggest a single factor, “helplessness” accounted for 29% of the variance (see Nowicki, 2018b, unpublished^2^). Nowicki (2018b, unpublished^2^) reports test–retest reliabilities ranging from 0.83 for 6 weeks to 0.56 for a year.

##### Discriminative validity

Duke and Nowicki (1974) investigated the relationship of ANSIE scores to social desirability. This was important because Rotter’s scale had been found to be significantly related to social desirability. Two samples of college students (*n* = 48, *n* = 68) were asked to complete the Marlowe–Crowne Social Desirability scale. Consistent with the requirements of discriminative validity, ANSIE scores were not related to scores from the social desirability measure [*r*(47) = 0.10; *r*(67) = 0.06]. Other studies reported in Nowicki (2018a, unpublished^1^) also have found ANSIE scores to be unrelated to social desirability scores.

##### Construct validity

Basically, the philosophy of construct validation implies that a new measure of a construct should show a significant relationship with well-established measures of that construct. An example of such a procedure is the correlating of a new measure of intelligence with the Stanford–Binet or with the Wechsler scales. If, however, the authors of a new measure assume the new measure adds something unique or measures the construct more accurately than the established measure, then the resulting relationship with the established measure should be somewhat less than identity. This is important to our present purpose. Since Rotter and others who have used his scale have amassed a large amount of data consistent with theoretical predictions from social learning theory, favorable comparison with this scale is indicated. It is predicted, therefore, that if the ANSIE scale is measuring the same construct as the Rotter scale the two should be significantly related. However, if the ANSIE is accounting for a unique portion of variance, then correlations between the measures should be positive, but should fall in the moderate range.

To assess the association between the ANSIE and the Rotter scales, Nowicki and Duke (1974) administered both scales to two college and community adult samples. In all three samples, the correlations between the two measures were significant and consistent with requirements [*r*(47) = 0.68, *p* < 0.01; *r*(37) = 0.48, *p* < 0.01]. These results are consistent with the contention that these two measures are assessing the same construct, but not in an identical manner. Nowicki, (2018b, unpublished^2^) reports that results of three other studies found ANSIE and Rotter scores correlated in the 0.50s.

Nowicki (2018b, unpublished^2^) reports studies whose results show ANSIE scores being related to participants’ race and socio-economic level in similar ways as Rotter’s scores with externality higher in non-white populations and lower socio-economic levels. The same pattern is true regarding evidence of psychological difficulties; externality is higher in those with psychiatric diagnoses, anxiety, and depression. Finally, similar to Rotter’s results, in a number of studies ANSIE test scores have been related to achievement as measured by standardized test scores and grade point average.

The ANSIE appears to meet the minimal requirements necessary for its use as an appropriate measure of LOC in adults. Further work is reported in Nowicki and Duke (1983), Nowicki (2016) and Nowicki (2018a, unpublished^1^).

The ANSIE scale used in the present study was developed specifically for ALSPAC; it comprised 12 questions taken from the original 40 question scale. The ANSIE was chosen over health-related scales because it was a generalized scale consistent with Rotter’s definition and has the potential to relate to a wider range of outcomes. This shortened ANSIE was validated on a sample of 135 pregnant women prior to use in ALSPAC. It was administered within self-completion questionnaires posted to the mothers during pregnancy, and subsequently 6 and 18 years later. In parallel, during pregnancy and 6 years post-delivery, the mothers were sent questionnaires for their partners to complete with identical LOC questions. When the study offspring were 20 years old, fathers were invited to a clinic, where they responded to a computerized questionnaire which included the identical set of LOC questions. Scores were computed by adding the number of external type answers; they ranged from 0 to 12 with higher scores indicating greater externality.

#### Children’s Locus of Control: Children’s Nowicki and Strickland Internal, External Scale (CNSIE, Nowicki and Strickland, 1973)

The children’s LOC measure used in the present study was an adaptation of the CNSIE (Nowicki and Strickland, 1973). The CNSIE has been used in over a 1000 studies that have provided data supportive of its construct validity (Nowicki 2018b, unpublished^2^). The Nowicki and Strickland Internal–External control scale is a paper and pencil measure of the LOC measure consisting of 40 questions answered by marking either the yes or no place next to the question. The final form of the scale derived from work which began with the construction of items (*n* = 102) based on Rotter’s definition of the internal–external control of reinforcement dimension. The items described reinforcement situations across areas such as affiliation, achievement, and dependency. School teachers helped in the construction of the items. The goal of such item construction was to make the items readable at the third-grade level yet appropriate for older students. To accomplish such a goal, the 102 items along with Rotter’s definition of the LOC dimension were given to a group of clinical psychology staff members (*n* = 9) who were asked to answer the items in an external direction. Items were dropped on which there was no complete agreement among the judges. This left 59 items which made up the preliminary form of the test. The 59-item form of the test was then given to a sample of children (*n* = 152) ranging from third through ninth grades. Means for this testing ranged from 19.1, *SD* = 3.86 at the third grade to 11.6, *SD* = 4.26 at the ninth grade with higher scores associated with an external orientation. Controlling for IQ, internals performed significantly better than externals on achievement test scores [*t*(48) = 3.78, *p* < 0.05]. Test–retest reliabilities for a 6-week period were *r*(98) = 0.67, *p* < 0.05, for the 8 to 11 year old group and *r*(54) = 0.75, *p* < 0.05, for those in the 12 to 15 year old group,

The 40-item scale was administered to children from the third through the 12^th^ grade to obtain reliability estimates, demographic measures and construct validity information. The sample consisted of 1017 elementary and high school students most of whom were Caucasian. All schools were in a county bordering a large metropolitan school system.

Nowicki and Strickland (1973) present biserial item correlations for males and females at the third, seventh, and 10th grades. The item-total relations are moderate but consistent for all ages. They also reported estimates of internal consistency via the split-half method, corrected by Spearman–Brown *r*(99) = 0.63 (grades 3, 4, 5); *r*(117) = 0.68 (grades 6, 7, 8); *r*(125) = 0.74 (grades 9, 10, 11); *r*(54) = 0.71 (grade 12). The reliabilities may be considered satisfactory because the items are not arranged according to difficulty. Since the test is additive and items are not comparable, the split-half reliabilities tend to underestimate the true internal consistency of the scale. Nowicki (2018b, unpublished^2^) includes internal consistency estimates from other studies that range from 0.60 to 0.70.

Nowicki (2018b, unpublished^2^) reports results of factor analyses of children that suggest the scale has a general “helplessness” factor of about 0.30. Nowicki and Strickland (1973) reported test–retest reliabilities sampled at three grade levels, 6 weeks apart; *r*(99) = 0.63 for third graders, *r*(117) = 0.66 for seventh graders, and *r*(125) = 0.71 for the 10th graders. Nowicki (2018b, unpublished^2^) reported the results of several other studies showing that test–retest correlations ranged from 0.67 over 6 weeks to 0.63 for 9 months.

##### Discriminative validity

A prime goal of those who construct LOC scales is to keep social desirability at a minimum. Nowicki and Strickland (1973) reported non-significant correlations between LOC scores and social desirability for subjects in grades 3–12. Nowicki (2018b, unpublished^2^) reported results of other studies that also found non-significant associations between CNSIE scores and social desirability. Further data are presented by Nowicki and Duke (1983) and Nowicki (2018b, unpublished^2^).

##### Construct validity

In terms of convergent validity support for the CNSIE, Nowicki and Strickland (1973) reported data showing significant but moderate relations between the CNSIE and other measures of LOC such as the Intellectual Achievement Responsibility and the Bialer-Cromwell scales. If a measure of a construct such as LOC has been found to be related to other variables in a theoretically consistent fashion, then the measure gains some degree of construct validity for the CNSIE. Nowicki and Strickland (1973) reported externality on the CNSIE was associated in a theoretically consistent manner with lower as opposed to upper social class and non-white as opposed to white participants.

In addition, results of studies support the theoretical assumption that internality is associated with higher and externality with lower academic achievement as well as to those behaviors associated with academic achievement, such as persistence. For example, Nowicki and Strickland (1973) reported significant correlations between internality and higher academic achievement for children from grades three through 12. Others (see Nowicki, 2018b, unpublished^2^) have confirmed the internality, achievement association not only in American but in Danish, Hungarian and Mexican children as well. Nowicki (2018b, unpublished^2^) reported that results from six studies found that internals persisted longer on tasks than externals. Finally, Nowicki (2018b, unpublished^2^) summarized research that showed that externality was associated with a variety of psychological and physical difficulties. Additional support for the construct validity of the CNSIE can be found elsewhere (Nowicki and Duke, 1983; Nowicki, 2016; Nowicki, 2018b, unpublished^2^).

The CNSIE form used in the present study originated from an administration of the 40-item test to a sample of 120 8 year-old-children and the 12 items with the best item-total correlation were chosen for inclusion in the final form administered to ALSPAC children when they were tested at 8 years of age. The questions were read aloud to the child by the examiner to eliminate variance due to reading ability. The child was asked to respond with a yes/no answer. The tester made clear that there were no right or wrong answers and the items reflected how people thought and felt about different things. A similar scale was sent to the study children in a self-completion questionnaire at age 16. Scores were computed adding the number of external type answers; they ranged from 0 to 12 with higher scores indicating greater externality.

### Statistical Analyses

Data were used as continuous when calculating correlation coefficients, and as binary when comparing external with internal orientation. For these analyses, an external LOC (ELOC) was defined as a score greater than the median and an internal LOC (ILOC) as equal to, or less than, the median. We chose to dichotomize both the parent and child data into ELOC and ILOC to facilitate easier interpretation of results. Because of the likelihood of collinearity, stepwise logistic regression was used with p to enter of 0.10, rather than multiple linear regression which assumes a linear association. Analyses were repeated for boys and girls separately. Pseudo-R^2^ was used as a measure of Goodness-of-fit (GOF).

## Results

The basic data for LOC distributions at each time point are shown in Table 1. For parents, externality was defined as having a LOC score above the median (4, 4, and 3 for mothers in pregnancy and 6 and 18 years later; and for fathers it was 3 at each time point). The offspring’s scores formed approximately normal distributions with medians of 6 at age 8, and 3 at 16 years.

**Table 1:** The means, standard deviations, and medians for the study parents at three-time points, and for the offspring at two time points measured from birth.

Individual	Time-point	*N*	Mean	*SD*	Median
Mother	Pregnancy	12604	4.37	2.11	4
	+ 6 years	8633	3.83	1.99	4
	+18 years	3758	3.48	2.01	3
Father	Pregnancy	8738	3.60	2.30	3
	+6 years	4507	3.28	2.06	3
	+20 years	1855	2.83	1.86	3
Offspring	Age 8 years	6374	5.99	2.08	6
	Age 16 years	4770	3.20	2.12	3

*SD, standard deviation.*

### Correlations Between Measures of LOC for the Individual

For 3487 mothers the LOC score was available at each of the three-time points. Correlation coefficients are presented here as they have the advantage of showing the relationships between the LOC measures between the different family members as well as over time. They were 0.55 and 0.54 for comparisons of pregnancy LOC with those 6 and 18 years later respectively. Similarly, the correlation between the measures of the mother at 6 and 18 years was strong (0.56). Although there were fewer fathers with measures at the three-time points (*n* = 1176), the correlations were equivalent to those found above for the mothers at 0.55, 0.52, and 0.55, respectively. In contrast the correlation between the child’s LOC measures at ages 8 and 16 were weak (0.22).

### Correlations Between Parents’ and Child LOC Measures

The correlations between parental LOC scores at the three ages with the child’s LOC scores are shown in Table 2. In comparison with the correlations within the parent over time (where the values were strong at > 0.51), the correlations between each parent and child were weak, ranging between 0.14 and 0.20 for mothers and 0.14 and 0.19 for fathers.

**Table 2:** Correlation coefficients between parents and children.

Individual	Time point	Child at 8 years	Child at 16 years	*N*
**Mother**				1859
	Pregnancy	0.166	0.169	
	6 years	0.142	0.160	
	18 years	0.171	0.203	
**Father**				726
	Pregnancy	0.193	0.157	
	6 years	0.160	0.189	
	20 years	0.142	0.143	

Examination of results for boys and girls separately indicated that correlations between maternal LOC and 16-year-old children tended to be slightly higher (range: 0.18 to 0.20) than found for the 8-year-olds, whereas the correlations between paternal LOC pregnancy score tended to be higher with child LOC at 8 (Table 2).

### Contributions of Parental Externality to Child Externality

Ways in which the binary estimates of the externality of the parents contribute toward the externality of the child are shown in Table 3. When all children are considered together, the externality of each parent at each of the two antecedent time points can be seen to be independently associated with the externality of the child. However, if the factors are considered separately for boys and girls, the patterns of association tend to differ: for boys there is no longer an association with paternal ELOC at 6 years; for girls, there is no longer an association with maternal ELOC in pregnancy, and the strongest association is with the fathers’ ELOC at 6 years.

**Table 3:** Stepwise logistic regression analyses to determine whether specific parental externalities were independently associated with externality of the 8 year old child.

Individual time point	Unadjusted			Adjusted		
	*n*	OR[95%CI]	*p*	*n*	OR[95%CI]	*p*
**All children**						
M – pregnancy	5902	1.46[1.31,1.62]	^∗∗∗∗^	2620	1.31[1.09,1.57	^∗∗^
M – 6 years	5227	1.48[1.32,1.67]	^∗∗∗∗^	2620	1.23[1.01,1.49]	^∗^
F – pregnancy	4403	1.64[1.45,1.85]	^∗∗∗∗^	2620	1.41[1.18,1.70]	^∗∗∗^
F – 6 years	2958	1.55[1.33,1.80]	^∗∗∗∗^	2620	1.30[1.08,1.55]	^∗∗^
**Boys**						
M – pregnancy	2960	1.45[1.24,1.68]	^∗∗∗∗^	1958	1.26[1.03,1.55	^∗^
M – 6 years	2660	1.46[1.23,1.72]	^∗∗∗∗^	1958	1.30[1.05,1.61]	^∗^
F – pregnancy	2202	1.59[1.34,1.90]	^∗∗∗∗^	1958	1.45[1.20,1.76]	^∗∗∗^
F–6 years	1503	1.40[1.13,1.73]	^∗∗^		DNE	
**Girls**						
M – pregnancy	2942	1.47[1.26,1.70]	^∗∗∗∗^		DNE	
M – 6 years	2567	1.50[1.27,1.78]	^∗∗∗∗^	1287	1.29[1.00,1.66]	^∗^
F – pregnancy	2201	1.69[1.42,2.00]	^∗∗∗∗^	1287	1.39[1.08,1.80]	^∗^
F – 6 years	1455	1.73[1.39,2.14]	^∗∗∗∗^	1287	1.58[1.22,2.04]	^∗∗∗^

*GOF for each analysis = 2.13 for all children, 1.51 for boys and 2.38 for girls. DNE, did not enter; F, father; M, mother; p-values: ^∗^ < 0.05; ^∗∗^ < 0.01; ^∗∗∗^ < 0.001; ^∗∗∗∗^ < 0.0001.*

### Contributions of Parental Externality to Adolescent Externality

Unadjusted odds ratios provide the difference between the straightforward results for external compared with internal individuals; adjusted odds ratios compute the difference after taking account of the other factors that may explain the relationship. For the risk of the 16-year-old being externally oriented, we first present the unadjusted odds ratios [95% confidence intervals], then the results of stepwise logistic regression offering the four parent measures (Adjustment A in Table 4), followed by the results of offering the adolescents’ own externality at age 8 (Adjustment B). Each of the five unadjusted measures were significantly associated with the adolescents’ externality; on adjustment A maternal ELOC in pregnancy dropped out, and in adjustment B paternal pregnancy ELOC also failed to enter – leaving the final model with the parental measures at 6 years, together with the child’s own measure at 8. Slightly different results were found for girls when considered on their own: maternal ELOC in pregnancy entered instead of the measure at 6 years (Table 4).

**Table 4:** Stepwise logistic regression analyses to determine whether specific parental externalities were independently associated with externality of the 16 year old offspring, first analyzing just for parental ELOC (model A), and then additionally for the child’s ELOC at age 8 (model B).

Individual time point	Unadjusted			Adjusted (A)		Adjusted (B)	*B*
	*n*	OR[95%CI]	*p*	OR[95%CI]	*p*	OR[95%CI]	*p*
**All children**							
M – pregnancy	4441	1.65[1.45,1.87]	^∗∗∗∗^	DNE		DNE	
M – 6 years	4033	1.69[1.47,1.94]	^∗∗∗∗^	1.59[1.30,1.94]	^∗∗∗∗^	1.43[1.14,1.79]	^∗∗^
F – pregnancy	3473	1.54[1.34,1.77]	^∗∗∗∗^	1.26[1.03,1.54]	^∗^	DNE	
F – 6 years	2485	1.75[1.48,2.08]	^∗∗∗∗^	1.54[1.26,1.88]	^∗∗∗∗^	1.56[1.28,1.92]	^∗∗∗∗^
Child at 8	3283	1.79[1.54,2.06]	^∗∗∗∗^	–		1.58[1.29,1.93]	^∗∗∗∗^
				(*n* = 2227; GOF = 2.32)		(*n* = 1818; GOF = 2.51)	
**Boys**							
M *–* pregnancy	1826	1.45[1.18,1.79]	^∗∗∗^	DNE		DNE	
M – 6 years	1707	1.59[1.28,1.98]	^∗∗∗∗^	1.61[1.19,2.18]	^∗∗^	1.83[1.28,2.61]	^∗∗∗^
F – pregnancy	1452	1.46[1.17,1.83]	^∗∗∗^	DNE		DNE	
F – 6 years	1083	1.88[1.44,2.44]	^∗∗∗∗^	1.75[1.34,2.30]	^∗∗∗∗^	1.75[1.28,2.40]	^∗∗∗^
Child at 8	1377	1.77[1.41,2.23]	^∗∗∗∗^	–		1.56[1.14,2.14]	^∗∗^
				(*N* = 1064; GOF = 2.26)		(*N* = 793; GOF = 3.66)	
**Girls**							
M – pregnancy	2615	1.74[1.48,2.04]	^∗∗∗∗^	1.38[1.07,1.78]	^∗^	1.43[1.09,1.89]	^∗^
M – 6 years	2326	1.74[1.46,2.09]	^∗∗∗∗^	1.29[0.99,1.70]	(^∗^)	DNE	
F – pregnancy	2021	1.57[1.31,1.88]	^∗∗∗∗^	DNE		DNE	
F – 6 years	1402	1.66[1.33,2.07]	^∗∗∗∗^	1.56[1.24,1.95]	^∗∗∗^	1.47[1.13,1.92]	^∗∗^
Child at 8	1906	1.77[1.47,2.14]	^∗∗∗∗^	–		1.63[1.25,2.12]	^∗∗∗^
				(*N* = 1372; GOF = 1.97)		(*N* = 1028; GOF = 2.48)	

*DNE, did not enter; F, father; M, mother; p-values: (^∗^) < 0.10; ^∗^ < 0.05; ^∗∗^ < 0.01; ^∗∗∗^ < 0.001; ^∗∗∗∗^ < 0.0001.*

## Discussion

The following summary of the results provides a foundation for the discussion to follow. Using a comparison of correlation coefficients, the present study found: (1) there was stronger evidence for continuity of parents’ LOC over time than there was for their offspring; (2) the correlations between the parents’ LOC and that of their offspring were significant but low at both 8 and 16 years of age. Further analysis categorizing external from internal individuals showed that: (3) each external parent was associated with an independent increased risk of their 8-year-old offspring being external, but the increased risk was <50%; (4) each parent who was external when the child was aged 6 was independently associated with the child’s LOC at age 16; (5) the external child at age 8 was at increased risk of being external at age 16 independent of the contribution of their parents’ LOC; (6) there were differences between the sexes in the patterns of association – *mothers with an external orientation at 6 years were associated with the externality of their sons* at *both* age 8 and age 16 while for the fathers *externality at 6* was associated with *their daughters’ externality* at both age 8 and age 16; (7) in addition, for predicting *boys’ externality at 8*, the loci of control of both parents were significantly associated, while for predicting *girls’ externality*, the external outlook of fathers’ prenatally and of mothers at age 6 were associated; (8) finally, for boys’ *externality at age 16*, fathers’ externality at age 6 and the boys’ own externality at age 8 were independently associated; while for *girls’ externality at age 16*, it was mothers’ prenatal externality as well as the girls’ externality at age 8 that were the significant predictors.

### Parent and Child Locus of Control

Parent LOC was associated with their children’s LOC, but only in a very limited manner at either point in time, regardless of which pairings were used, be they of same gender, mother prominent, or father prominent. Correlations ranged from near zero to the low 0.20s indicating that other factors besides parent LOC are involved in determining children’s orientation.

Although modest in size there was a pattern of differences depending on the sex of the child and the gender of the parent that, to a certain extent, supported the dominant parent prediction, but *across* genders not *between* them. *Mothers’* externality at 6 years was independently associated with *boys’ externality* at both age 8 and age 16 while *fathers’* externality at 6 years was significantly related to girls’ LOC at both testing periods. The finding of significant, cross parent, child LOC associations suggests that parents of the opposite sex of the child may be having an impact on establishing LOC orientations in their children either by more clearly modeling LOC behaviors or through interactions with the child that reinforce the child’s own LOC. Observational research of actual parent child interactions could provide evidence to support these or other possibilities (see Carton et al., 1996).

Identifying antecedents of children’s LOC appears to be more complex than children modeling the LOC of their parents. The relatively low parent, child LOC associations suggests that researchers need to search for other possible intervening variables involved in the development of LOC. Rotter (1966) and Lefcourt (1976) both theorized that the accurate perception of connections between behaviors and outcomes could be learned: (1) through the use of contingent reinforcement in which the behavior outcome sequence is reinforced by others at the time of its occurrence, or (2) via modeling in which children have opportunities to observe internal or external behavior in their parents and develop internal or external expectancies of their own in response. However, data concerning the presence or absence of contingent reinforcement sequences and/or the degree to which parents actually display behaviors associated with their LOC orientation are lacking in past studies and in the present one as well.

To evaluate the modeling hypothesis, it would be important to know how often and how well parents display clear and explicit examples of internality or externality. Operationalizing behavioral attributes of LOC such as delay of gratification, responsibility, persistence, resistance to coercion or information gathering would be helpful in this effort. Up to now, most researchers have simply assessed parent LOC and assumed they raised their children consistent with their orientation and children somehow “pick up” parents’ tendency to behave in internal or external ways and in turn are motivated to model them. The present study found a significant but small association between parent and child LOC, suggesting there is much more to learn about what else parents do to facilitate the learning of internal/external control expectancies in their offspring.

After reviewing the literature concerning antecedents of LOC, Carton and Nowicki (1994) recommended that researchers gather more observational information about how parents interact with their children across the full range of control expectancies. They suggested use of the concept of “goodness of fit” as one way to better gauge the learning environment of children. For example, certain types of children’s temperament may “fit” better with internal as opposed to external parent LOC. The better the “fit” the better are conditions for learning the connection between behavior and consequences. On the one hand, for example, it could be predicted that children with temperaments that would be easier for parents to deal with would be more likely to learn the behavior consequence sequences necessary for developing internality. On the other hand, it also could be predicted that children whose temperaments command more time, attention, and focus from parents may be the ones to elicit more behaviors associated with the parents’ LOC resulting in a higher parent, child LOC association. More research is needed to clarify parents’ role in children’s LOC.

### Stability of Locus of Control Across Time: Adults

Locus of control in adults between early to middle adulthood, appears to be relatively stable from before the child was born to when the child was aged 6 and 18–20. Correlations in the 0.50s for both mothers and fathers across all measurement points suggest consistent stability that didn’t seem differentially affected by the birth of the child. Correlations in the 0.50s, though significant, still leave considerable variance unaccounted for, and indicate that many women and men changed their LOC orientation over time. It would be helpful to know what events were associated with stability and changes toward internality or externality. Such information could provide valuable insights into how control expectancies are learned, which in turn could be used to develop interventions to change LOC.

Researchers have identified some factors associated with change in adult LOC as measured by the present tests. Nowicki et al. (2018a) found parent LOC change over 6 years to be associated with the type and number of stressful experiences involving *relationships*, *health*, and *finances*. These findings are consistent with those found using other LOC tests. For example, Elkins et al. (2017) found that long term difficulties rather than single events appeared to be associated with changes in LOC as measured by items from Pearlin and Schooler’s Mastery module. In addition, other research (Nowicki et al., 2018b) found that changes toward greater parent externality were associated with children having more teacher reported difficulties, while transitions to greater parent internality were characterized by fewer ones.

### Stability of Locus of Control Across Time: Children

Children’s LOC scores are less stable than those of their parents. Children’s emotional, physical, and cognitive abilities are changing more quickly and extensively than their parents. Based largely on past cross-sectional findings, children’s LOC appeared to become more internal during childhood and into adolescence. However, there are scant data from longitudinal studies to confirm this trajectory within children over time. Although the mean LOC scores of children was more internal at age 16 than at age 8, the correlations between the two ages is only 0.23, revealing significant intra-and inter-child change between the two testing times. In contrast to cross-sectional data which suggest stable movement toward internality during childhood, our longitudinal data suggest considerable volatility and change in children’s LOC between the ages of 8 and 16. Because of its relative instability, finding factors associated with stability and change in children’s LOC are important and should be identified.

### Limitations

Although this study is limited by the gaps between measures of LOC, it is unique in having longitudinal data with a large population sample, utilizing trios of mothers, fathers, and their children. The major limitation is a greater loss of external than internal participants over time.

## Conclusion

The present study is among the first to establish consistency of adult LOC scores beginning before children were born and extending for two decades, and child LOC extending from childhood into adolescence using standardized and construct valid tests tied to Rotter’s definition. Adults’ LOC scores are significantly more consistent than children’s. We also found a significant, but small, correlation between parent and child LOC and suggested that other factors, such as child temperament and time parents and children interact, may be associated with the development of control expectancies. We urge future researchers to focus on gathering observational information concerning parent, child interactions, and operationalizing parent behavior that reflects their control expectancies.

## Author Contributions

SN had the idea. JG planned the analyses. GE and SG undertook the statistical analyses. JG and SN wrote the first draft. All authors contributed equally to writing later drafts, checking, and editing.

## Conflict of Interest Statement

The authors declare that the research was conducted in the absence of any commercial or financial relationships that could be construed as a potential conflict of interest.

## Footnotes

1 Nowicki, S. (2018a). *A Manual for the Adult Nowicki Strickland Internal External Scale (ANSIE)*. Georgia: Emory University.

2 Nowicki, S. (2018b). *A Manual for the Children’s Nowicki Strickland Internal External scale (CNSIE)*. Georgia: Emory University.
